# Chemical transdifferentiation of somatic cells to neural cells: a systematic review

**DOI:** 10.31744/einstein_journal/2024RW0423

**Published:** 2024-11-12

**Authors:** Paulo Victor Visintin, Bruna Lancia Zampieri, Karina Griesi-Oliveira

**Affiliations:** 1 Hospital Israelita Albert Einstein São Paulo SP Brazil Hospital Israelita Albert Einstein, São Paulo, SP, Brazil.

**Keywords:** Transdifferentiation, Direct conversion, Small molecule, Chemical cocktail, Neural cells

## Abstract

**Introduction:**

Transdifferentiation is the conversion of a specific somatic cell into another cell type, bypassing a transient pluripotent state. This implies a faster method to generate cells of interest with the additional benefit of reduced tumorigenic risk for clinical use.

**Objective:**

We describe protocols that use small molecules as direct conversion inducers, without the need for exogenous factors, to evaluate the potential of cell transdifferentiation for pharmacological and clinical applications.

**Methods:**

In this systematic review, using PRISMA guidelines, we conducted a personalized search strategy in four databases (PubMed, Scopus, Embase, and Web Of Science), looking for experimental works that used exclusively small molecules for transdifferentiation of non-neural cell types into neural lineage cells.

**Results:**

We explored the main biological mechanisms involved in direct cell conversion induced by different small molecules used in 33 experimental *in vitro* and *in vitro* transdifferentiation protocols. We also summarize the main characteristics of these protocols, such as the chemical cocktails used, time for transdifferentiation, and conversion efficiency.

**Conclusion:**

Small molecules-based protocols for neuronal transdifferentiation are reasonably safe, economical, accessible, and are a promising alternative for future use in regenerative medicine and pharmacology.

## INTRODUCTION

A pioneering study carried out by Takahashi et al.^(1)^ showed the possibility of inducing a somatic cell to return to a pluripotent stage, providing a new perspective on the reversibility of the cell differentiation process. Through ectopic expression of four transcription factors (TFs), Oct3 / 4, Sox2, Klf-4, and c-Myc (OSKM; also called Yamanaka factors), murine fibroblasts were reprogrammed into pluripotent stem cells with physiological potential resembling embryonic stem cells (ESCs). The generated so-called induced pluripotent stem cells (iPSCs) can self-renew into pluripotent cells or differentiate into somatic cells from any of the three embryonic layers.^(1)^ The success of cell reprogramming has opened new avenues for both basic research and regenerative therapy.^(2)^ As iPSCs are isogenic to the individual donor, they are a good biological model for *in vitro* studies of diseases whose tissue of interest is difficult to access or whose acquisition is too invasive, or both, as in the case of neurological diseases. Thus, the use of cell reprogramming techniques, which allow the generation of disease-specific models, makes it possible to study disease pathogenesis and identify novel therapeutic targets through drug development and screening against a specific donor’s genetic background.^(2,3)^

Given the immunological rejection events and ethical issues related to the use of ESCs, the advent of iPSCs technology has been seen as a promising alternative for personalized regenerative medicine for several conditions, including incurable central nervous system (CNS) diseases.^(2,4)^ Although iPSCs generation has good prospects, it is a laborious and expensive process, and the successful reprogramming of somatic cells is complex and not always predictable.^(5)^A potential pitfall is that their unlimited ability to differentiate and self-renew into any tissue poses a tumorigenic risk, restricting their potential clinical use.^(6-8)^

**Figure 1:**
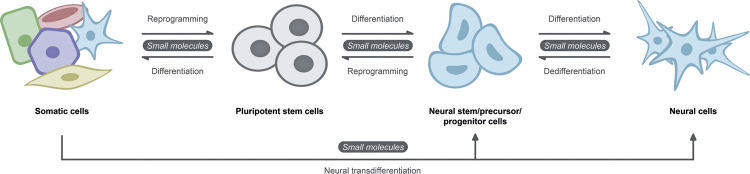
Cell fate conversions by chemical induction protocols Small molecules are used in reprogramming protocols, in which somatic cells are converted to a pluripotent state, and vice versa, for cell differentiation. A promising application of small molecules is in neural transdifferentiation protocols that bypass the pluripotent state, which provides a faster way to generate cells of interest with reduced tumorigenic risk for clinical use. Source: The authors.

As an alternative, cell transdifferentiation, in which somatic cells are directly converted into another somatic lineage or multipotent stem cells, thereby bypassing the pluripotent stage, enables a faster and safer way to obtain the target cell type(s)^(9,10)^(Figure 1). In this sense, considering the nervous tissue, transdifferentiation can be applied to obtain cells in the final stage of differentiation, such as induced neuron cells (iNs),^(11)^or for the generation of multipotent neural stem cells such as induced neural progenitor cells (iNPCs) or induced neural stem cells (iNSCs).^(12)^

Initial transdifferentiation protocols for neural cells used the same strategy as the original cell reprogramming protocol developed by Takahashi et al. that is, ectopic expression of different sets of TFs was achieved through viral transduction in non-neuronal cells.^(13,14)^However, in the context of clinical applications, the transduction of viral vectors does not offer adequate safety to patients because random integration of the vectors into host cell genomes can lead to genetic alterations.^(15)^ To circumvent this issue, transdifferentiation can also be induced by exogenous TFs delivered to the cells using integration-free viruses,^(16)^plasmid DNA^(17)^ (which have a reduced, but not null, risk of insertion into the genome), synthetic mRNA,^(18)^epigenetic modifiers,^(19)^ or recombinant proteins.^(20)^

One promising approach is to promote transdifferentiation using low-molecular-weight organic compounds called small molecules (SMs), which are organic bioactive compounds with a molecular weight of less than 900 Da and an average size of 10^-9^ m.^(21)^ Small molecules were initially used to achieve a higher TF-mediated conversion efficiency.^(22)^However, because of their gene expression regulatory capacity, the use of SMs alone successfully enables the conversion of different somatic cell lineages into many types of functional cells, including neural cells, cardiomyocytes, adipocytes, skeletal muscle cells, beta cells, cartilaginous cells, photoreceptor cells, and Leydig cells *in vitro*,^(23-25)^as well as stem cells such as NSCs, oligodendrocyte progenitor cells (OPCs), endoderm progenitor cells, and pluripotent stem cells.^(26,27)^

Although the mechanisms involved in this transcriptional landscape reprogramming have not yet been fully elucidated, some aspects are well-documented. It is known, for example, that SMs can modulate gene expression by regulating main cell signaling pathways, as well as acting in chromatin conformation, metabolic modulation, cytoskeleton activity, among other functions.^(28-31)^As a viral-free and genome integration-free approach, SMs not only show capabilities for generating specific cell types to be applied to disease modeling and cell transplantation but may also be used directly as drugs that can restore tissue *in vivo*.^(32,33)^

Compared to other reprogramming methods, transdifferentiation using SMs has several major advantages because they can be applied at flexible concentrations and in different cocktail combinations to achieve different cellular responses.^(33-35)^Moreover, the use of SMs allows for better temporary control over the effects triggered in cells, as this can be manipulated in a transitory and reversible manner.^(^^36^^)^ Additionally, SMs represent a more economical alternative to recombinant proteins. These properties render SMs safer and more efficient alternatives, particularly for use in clinical regenerative medicine.^(15,37,38)^

The advantages and feasibility of using SMs to induce different cell types across germ layers and lineages have been demonstrated, and it is particularly interesting to induce neural fate. Direct neural conversion involves permanent epigenetic changes in initial somatic cells to achieve the desired neural cell identity. This includes a combination of the activation of neural genes and repression of other genes related to non-neural cells.^(39)^In summary, the conversion of human somatic cells into iNSCs and iNs through SMs holds promise as a possible alternative treatment for diseases, including spinal cord injury, Huntington’s disease, and Alzheimer’s disease^(27,40-44)^ as well as for modeling nervous system disorders, including schizophrenia, autism spectrum disorder, Dravet syndrome, mild febrile seizures, and glaucoma.^(45)^

This systematic review addresses the current scenario regarding the use of SMs for the transdifferentiation of human and murine somatic cells to neural destinations, such as iNSCs, iNPCs, neurons, astrocytes, oligodendrocytes, and Schwann cells. We also describe some of the biological mechanisms involved in this type of cell conversion and their main limitations.

## METHODS

This systematic review was structured based on the Preferred Reporting Items for Systematic Reviews and Meta-Analyses (PRISMA) guideline (“The PRISMA 2020 statement: an updated guideline for reporting systematic reviews,” 2020).^(46)^

For a broad search of the specific subject of this review, we defined three keyword categories (Technique, Induction type, and Cell fate). In the preliminary step of the investigation, we built a list of terms with similar meanings that were frequently used in the area. For instance, for the category “Technique,” we found in the literature terms such as “transdifferentiation,” “direct conversion,” and “direct reprogramming.”. For the category “Induction type,” we defined terms such as “small molecule,” “chemical cocktails,” and “integration-free,” to specify studies with only chemical transdifferentiation approaches. Finally, in the category “Cell fate,” we searched for terms referring to neural lineages like “neurons,” “NPCs,” “NSCs,” and “neuroglia,” as this review is restricted to neural transdifferentiation. A literature search was performed in four different databases (PubMed, Scopus, Embase, and Web of Science) using keywords and the search strategies described in Table 1S, Supplementary Material; the search approach was adapted to each database according to their particularities. The literature search was conducted between March 18, 2021, and December 7, 2021, resulting in 482 distinct studies dating from 1987 to 2021.

As inclusion criteria, we considered studies that simultaneously: a) used direct cell conversion method (transdifferentiation); b) applied exclusively SMs as cell conversion inducers, without the use of vector-based exogenous gene expression approaches; c) used non-neural somatic cells as a source for neural transdifferentiation; d) obtained, as an outcome, neural cells characterized by morphological and neural gene expression analysis.

As an exclusion criteria, we rejected studies that: a) focused on other topics; b) used hard-to-access multipotent [such as: adipose tissue-derived stem cells (ADSCs), mesenchymal stem or stromal cells (MSCs), gingival mesenchymal stem cells (GMSCs), spermatogonial stem cells (SSCs), and muscle-derived cells (MDCs)] or pluripotent stem cells as a sourcing material; c) induced cell conversion from tumor cells; d) or used transient episomal delivery or TAT transduction system for induction as a method. Notably, the publication date of the study was not an exclusion criterion.

**Figure 2:**
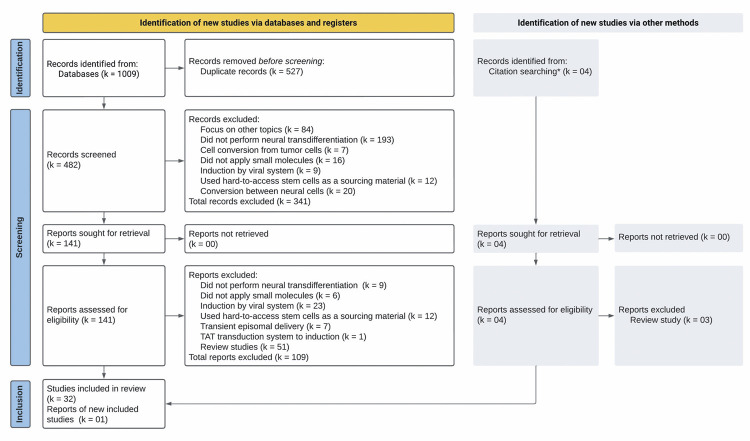
Flow diagram used to select eligible studies The PRISMA guideline is used to select the eligible studies. A literature search is performed in PubMed, Scopus, Embase, and Web of Science databases, resulting in 482 unique papers. After applying the inclusion and exclusion criteria to all records’ abstracts, 141 are retrieved for an integral content analysis step, resulting in 32 experimental studies selected for inclusion in this review. *In the citation search, we search for new studies cited in review studies derived from reports that were assessed for eligibility. One additional experimental study is included, resulting in a final list of 33 studies for this review.

A total of 33 eligible experimental studies conducted from 2014 to 2021 were included in this systematic review (Figure 2).

### Biological mechanisms of chemical transdifferentiation

**Figure 3:**
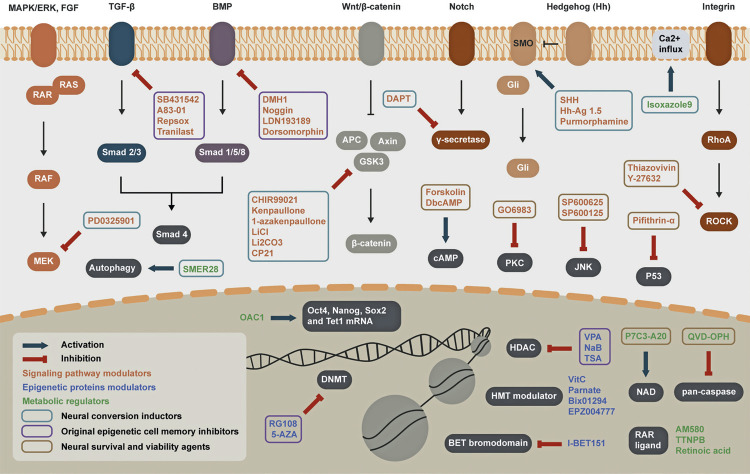
Biological mechanisms of neural transdifferentiation by small molecules During the neural transdifferentiation process, small molecules regulate gene transcription by modulating signaling pathways, epigenetic proteins, and metabolism. Source: The authors.

The efficiency of cell reprogramming, transdifferentiation, and differentiation depends on several factors, such as cell identity, cell cycle, and circadian and epigenetic status.^(47)^SMs, as modulators of different biological processes, can regulate gene transcription through four possible mechanisms, overviewed in figure 3: 1) modulation of signaling pathways, by activating or repressing signal transduction components to regulate the activity of transcription; 2) modulation of epigenetic proteins, regulating the activity of epigenetic complexes, indirectly contributing to transcriptional activation or repression; 3) metabolism regulation, adjusting cell state and altering the balance of protein-binding metabolites and epigenetic protein cofactors; 4) modulation of nuclear receptors, acting as agonists and antagonists to regulate nuclear receptor activity, thus directly modulating transcription.^(48)^Table 1 provides a list of SMs divided according to their mechanisms of action and the respective applications in which these molecules were used for neural transdifferentiation. Notably, although SMs that act as nuclear receptor modulators have been used in protocols to induce pluripotency,^(48-51)^no registry of their use for neural transdifferentiation has been found in the literature reviewed here; therefore, these will not be discussed.

**Table 1:** Small molecule functions and their application in neural cell transdifferentiation protocols

Class	Function	Small molecule	Neural cell lineages achieved in transdifferentiation protocols
Signaling pathway modulators	TGF-β inhibitor	SB431542	iNSC, iNPC, iNCC, iN, iA, iSC
		A83-01	iNSC, iNCC, iN, iGlN, iOPC, iA
		Repsox	iNSC, iNPC, iNCC, neural cells, iN, DA iN, iA
		Tranilast	iNPC
	GSK-3β inhibitor	CHIR99021 (CHIR)	iNSC, iNPC, neural cells, iNCC, iN, iGlN, iPNSN, iOPC, iA
		Kenpaullone	iN, DA iN
		1-azakenpaullone (1-AZA)	iNSC
		Lithium chloride (LiCl)	iNPC
		Lithium carbonate (Li2CO3)	iNPC
		CP21	iSC
	MEK\ERK signaling inhibitor	PD0325901 (PD)	iNSC, iN, iGlN
	AMPK and BMP-1 receptor inhibitor	Dorsomorphin (DM)	iNCC, neural cell, iN, iGlN
	BMP inhibitor	noggin	iSC
		LDN193189 (LDN)	iNSC, iNCC, iN, iGlN, iPNSN, iOPC
		DMH1	iN
	cAMP activator	Forskolin (FSK)	iNPC, iNCC, neural cell, iN, DA iN, iGlN, iA
		Db-cAMP	iSC
	Smoothened agonist	Purmorphamine (PUR)	iNSC, iN, DA iN, iGlN
		Hh-Ag 1.5	iNSC, iOPC
		Human Sonic Hedgehog (Shh)	iNSC, DA iN, iPNSN, iOPC, iSC
	γ-secretase inhibitor	DAPT	iNSC, iGlN, iPNSN
	JNK inhibitor	SP600625	neural cell, iN
		SP600125	iNPC, neural cell, iN
	ROCK inhibitor	Thiazovivin (Tzv)	iNSC, neural cell, iOPC
		Y-27632	iNCC, neural cell, iN, iGlN
	PKC inhibitor	GO6983	iNPC, neural cell, iN
	p53 inhibitor	Pifithrin-α	iN
Epigenetic proteins modulators	HDAC inhibitor	Valproic acid (VPA)	iNSC, iNPC, iNCC, iN, DA iN, neural cell, iA
		Sodium butyrate (NaB)	iNPC, iN
		Trichostatin (TSA)	iNPC, iNCC
	HMT modulator	Ascorbic acid (VitC)	iNSC, iNPC, iN, DA iN, neural cells, iSC
		Tranylcypromine (Parnate)	iNSC, iNCC, iN, iOPC, iA
		Bix01294	iNSC
		EPZ004777 (EPZ)	iNCC
	DNMT inhibitor	RG108	iNSC, iNCC, Neural cells, iGlN, iOPC
		5-azacytidine (5-AZA)	iNSC, iNCC
	BET bromodomain inhibitor	I-BET151	iN
Metabolic regulators	Autophagy metabolism activator	SMER28	iNSC, iNCC, iOPC
	RAR ligand	Retinoic acid (RA)	iNSC, iNCC, iN, iOPC
		TTNPB	iN
		AM580	iNCC
	Ca2+ influx activator	Isoxazole9 (ISX9)	Neural cell, iN, iGlN
	Oct4, Nanog, Sox2 and Tet1 mRNA level activator	OAC1	iA
	Pan-caspase inhibitor	QVD-OPH	iN
	NAMPT-relevant pathway inductor	P7C3-A20	iGlN

Small molecule function: DNMT, DNA methyltransferase; HMT: histone methyltransferase; HDAC: histone deacetylase; LSD1: lysine-specific demethylase 1; PI3K: phosphoinositide 3-kinase; SHH: human sonic hedgehog; JNK: c-Jun N-terminal kinase; MAPK: mitogen-activated protein kinase; PKC: protein kinase C; LSD 1: lysine-specific demethylase-1. Neural cell lineages: iGlN: induced glutamatergic neuron; iOPC: induced oligodendrocyte progenitor cell; iN: induced neuron; DA iN: induced dopaminergic neuron; iA: induced astrocyte; iNCC: induced neural crest lineage cell; iNSC: induced neural stem cell; iNPC: induced neural progenitor cell; iSC: induced Schwann cell; iPNSN: induced peptidergic nociceptive sensory neuron.

### Signaling pathway modulators

#### Transforming growth factor-beta (TGF-β) pathway inhibitors

Several SMs used in neural transdifferentiation act as inhibitors of the growth factor-beta (TGF-β) pathway. The TGF-β pathway plays an important role in cell development as an epithelial-to-mesenchymal transition inductor. Thus, its inhibition can suppress the fibroblast gene expression program, consequently facilitating mesenchymal-to-epithelial transition (MET) and neuroectoderm specification.^(21)^Some of the TGF-β inhibitors commonly used are: SB431542 (SB) and A83-01, inhibitors of ALK5/4/7 receptors (TGFβR / ALK5/4/7i), which inhibits TGF-β-mediated activation of SMAD proteins;^(28,47)^RepSox (E-616452), a TGFβR-1 / ALK5i capable of replacing the TF SOX2 and improving the expression of NANOG;^(47)^and Tranilast, an inhibitor of receptors for platelet-derived growth factors and transient receptor potential vanilloid 2 channels.^(52)^

## MEK-ERK pathway inhibitor

When inhibited by the upstream TGF-β signaling pathway, MEK-ERK signaling induces different reprogramming steps and stimulates MET.^(53)^ PD0325901 (PD), a MEK\ERK signaling inhibitor, is often used to increase neuron-like cell conversion yield.^(53-55)^

## Glycogen synthase kinase 3 (GSK-3) pathway inhibitors

Glycogen synthase kinase 3 (GSK-3) induces B-catenin phosphorylation, targeting this molecule for degradation. As such, the inhibition of GSK-3 leads to the activation of the B-catenin/Wnt pathway, which is another important pathway involved in neuronal development that suppresses the mesenchymal phenotype and promotes MET.^(56,57)^CHIR99021 (CHIR) is a GSK3-blocking molecule that facilitates neuroectodermal differentiation via Wnt pathway activation.^(58)^ Other molecules with similar functions are kenpaullone, 1-azakenpaullone (1-AZA), lithium chloride (LiCl), lithium carbonate (Li_2_CO_3_), and CP21, which are used to induce neural progenitor cells and neural lineage cells.

## Bone morphogenic protein pathway inhibitors

Bone morphogenic protein (BMP) signaling is a fundamental signaling pathway during embryogenesis owing to its role in inducing mesoderm and endoderm differentiation.^(59)^Thus, its inhibition facilitates the induction of ectodermal differentiation and, consequently, neural fate. Attempts to use BMP signaling inhibitors, such as DMH1, LDN193189 (LDN), and particularly dorsomorphin (DM) and noggin, for neural transdifferentiation are mainly based on the extensively documented use of these molecules to promote neural differentiation from ESCs or iPSCs.^(60-62)^

## Sonic hedgehog pathway inhibitors

The sonic hedgehog (SHH) signaling pathway is another target for the modulation of neural transdifferentiation because of its role in ventral central nervous system development. Several agonists of the SHH pathway, such as purmorphamine (PUR), Hh-Ag 1.5, and SHH, have also been used to achieve neural cell fates different from those of somatic cells.^(56,63,64)^

## Other signaling pathway modulators

Some SMs also play a role in maintaining cell survival and preventing apoptosis during cell conversion, while modulating different pathways. Examples of these SMs are forskolin (FSK),^(47)^DbcAMP,^(24)^SP600625, SP600125,^(65)^thiazovivin (Tzv), and Y-27632.^(28)^Other pathway inhibitors commonly used to induce a neural fate are: DAPT, a gamma-secretase inhibitor that modulates the Notch pathway activity;^(55,63,66,67)^GO6983, a protein kinase C (PKC) inhibitor;^(43,68)^and pifithrin-α, a p53 inhibitor.^(53)^

## Epigenetic protein modulators

Several molecules with epigenetic modulation activity have been used in reprogramming protocols, as they have been found to increase the efficiency of iPSC generation in combination with the overexpression of different sets of TFs.^(69,70)^ Their role in such protocols is primarily attributed to their ability to promote an epigenetic state that facilitates the access of TFs to regulatory regions, thereby contributing to transcriptional profile changes. Owing to their recognized activity, many of these molecules have been tested using transdifferentiation protocols. The types of epigenetic protein modulators commonly used in neural transdifferentiation protocols are histone deacetylase inhibitors (HDACis), histone methyltransferase (HMT) modulators, and DNA methyltransferase (DNMT) inhibitors.

Histone deacetylase inhibitors mediates cell conversion through different mechanisms, such as TFs and histone deacetylation, or by regulating deacetylation, resulting in chromatin remodeling.^(71)^ Some examples of HDACis are valproic acid (VPA), a possible modulator of the mTOR signaling pathway;^(47,63)^sodium butyrate (NaB), which can upregulate the miR302/367 cluster;^(72)^and trichostatin (TSA), used to generate chemical-induced neural progenitor cells (ciNPCs) and induced neural crest lineage cells.^(73)^

Histone methyltransferase modulators transfer methyl groups from the cofactor s-adenosyl methionine to lysine and arginine residues of histones.^(71)^For instance, parnate is an inhibitor of the enzyme-specific lysine demethylase 1 (LSD1), which leads to H4K4 demethylation.^(28)^ Bix01294, a G9a HMTase inhibitor;^(54)^EPZ004777 (EPZ), a disruptor of the telomeric silencing 1-like (DOPTiL) inhibitor;^(74)^and ascorbic acid (VitC), an antioxidant capable of promoting histone or DNA demethylation,^(37)^ are other molecules that modulate HMT activity.

DNA methyltransferase inhibitors, such as 5-azacytidine (5-AZA)^(52)^and RG108, which block the active site of DNMT,^(37)^ belong to a class of molecules that suppresses the action of an enzyme family that catalyzes the methylation of cytosine to form 5-methylcytosine (5mC)^(29)^and promotes epigenetic regulation.

Bromodomain and extraterminal (BET) bromodomain inhibitors suppress bromodomain proteins by coupling histone acetylation with transcriptional regulation.^(75)^I-BET151, an example of an SM in this class, represses the original cell epigenetic memory, thus contributing to cell reprogramming.^(27)^

## Metabolic regulators

Metabolic regulators with neural induction properties include retinoic acid (RA) and other RAR ligands, which contribute to neurogenesis and neuronal differentiation by activating RA receptors.^(57)^ Quinolyl-valyl-O-methylaspartyl- [2,6-difluorophenoxy]-methyl ketone (QVD-OPH), a potent pan-caspase inhibitor that prevents caspase-dependent cell apoptosis^(68)^and P7C3-A20, an agent that stimulates NAMPT-relevant pathways and has been shown to induce neurogenesis and neuroprotection in neurons derived from fibroblasts are other examples.^(55)^Moreover, other SMs in this class include isoxazole9 (ISX9),^(68)^ OAC1,^(38)^AM580, and TTNPB.

Activation of the cellular autophagy pathway may also be closely linked to successful conversions, as suggested by studies showing that the transdifferentiation of HFFs to neural fates by SMs increases the expression of autophagy-related genes and leads to the activation of such a mechanism.^(66)^In accordance with this, a study on fibroblast transdifferentiation using SMER28, an autophagy metabolism modulator, reported an enhanced generation of Sox2+/Nestin+ cells.^(76)^

## Chemically induced neural stem or progenitor cells

Several protocols have been employed to induce the conversion of mouse and human somatic cells into neural cells (Table 2). For example, a cocktail containing CHIR, VPA, Bix01294, RG108, PD, VitC, and A83-01, without the exogenous expression of TFs, can directly convert mouse embryonic fibroblasts (MEFs) into iNSCs.^(54)^These iNSCs efficiently differentiated into astrocytes, oligodendrocytes, and functional neurons both *in vitro* and *in vivo*. Another study showed the transdifferentiation of MEFs into iNSCs employing the chemical cocktail M9 [CHIR, A83-01, LDN, RA, Hh-Ag1.5, RG108, SMER28, parnate, and basic fibroblast growth factor (bFGF)] after 10 days of induction.^(76)^ The iNSCs generated showed double-positive NSC markers Sox2+/Nestin+, as well as the capacity for differentiation and self-renewal *in vitro* and *in vivo* similar to primary NSCs. Functional tests showed that mature neurons derived from ciNSCs can fire action potentials. In addition, ciNSCs grafted into postnatal mouse pup cortices differentiated *in vivo* into Olig2+ oligodendrocytes, GFAP+ astrocytes, and NeuN+ mature neurons, with no tumor formation up to four weeks post-injection.

**Table 2:** Studies that used chemical induction-based protocols to generate neural cells from accessible non-neural cells

Author	Donor cells	Cell lineages achieved	Small molecules	Supplementation	Neural marker expression efficiency	Time	Phenotype analysis	Transcript analysis	Functional analysis	*In vivo* transplantation / conversion
Duan et al., 2019^(63)^	MEF	iNSC	CH, VPA, LDN, SB, DAPT, SHH, and PUR	FBS, N2, B27, bFGF, and EGF	Nestin+ (76.7%) and Sox2+ (44.2%)	10 days	ICC and FC	RT-qPCR	n/a	n/a
		iNSC -> iN	VitC, SHH, and RA	N2, B27, BDNF, GDNF, cAMP	Tuj1+ (58% ± 9%)and NeuN+	~40 days				
		iNSC -> iOL	VitC	NT-3, PDGF, N2, B27	GFAP+ (61% ± 14%)	~28 days				
		iNSC -> iA	VitC	CNTF, BDNF, GDNF	Olig2+ (53% ± 6%)	18 days				
Zheng et al., 2016^(77)^	MEF	iNSC	VPA, A83-01, Tzv, and PUR	EGF, FGF	Nestin+ (>80%)and Sox2+ (41%)	12 days	ICC	RT-qPCR	n/a	n/a
		iNSC -> iN	n/a	BDNF	Tuj1+ (35%)	7 days		RT-PCR	WCR	
		iNSC -> iOL	specific differentiation protocol	specific differentiation protocol	Olig2+ (60%) and O4+ (45%)				n/a	
		iNSC -> iA			GFAP+ (30%)					
Zhang et al., 2016^(76)^	MEF	iNSC	M9 (CH, LDN, A83-01, RA, Hh-Ag1.5, RG108, Parnate, SMER28, and bFGF)	EGF, FBS, BSA, N2, B27	Sox2+/Nestin+ (24.2-30.04%)	10 days	ICC and FC	RT-qPCR, RNA-seq, ChIP-seq, ChIP-qPCR	n/a	iNSCs can differentiate into mature neurons, oligodendrocytes, and astrocytes with no tumor formation up to 4 weeks post-injection.
		iNSC -> iN	M9, VitC, and db-cAMP	BDNF, NT3, and GDNF	Tuj1+ (~67.9%), Map2+, NeuN+, and Synapsin I+	10-20 days	ICC	RT-qPCR	WCR	
		iNSC -> iOL	RA, SHH, LDN, and db-cAMP	PDGF-AA, bFGF, T3, and NT3	O4+, MBP+, MAG+, and MOG+	11-17 days			n/a	
		iNSC -> iA	SHH, LDN, and db-cAMP	T3, NT3, and BMP4	Gfap+ (~16.5%) and S100b+	8-12 days				
Han et al., 2016^(54)^	MEF and TTF	iNSC	CH, VPA, Bix01294, RG108, PD, VitC, and A83-01	FBS, EGF, bFGF, FBS, N2, and Lif	Sox2+, GFAP+, Olig2+, and Gli2+	~4 weeks	ICC and ALP	RT-PCR, RTprofiler PCR	n/a	ciNSCs can differentiate into astrocytes, functional neurons, and oligodendrocytes *in vitro* and *in vivo*.
		iNSC -> iN	FSK, RA, and db-cAMP	FBS, N2, B27, BNDF, GNDF	MAP2+ (31-36%), Vamp2+, and NeuN+	4 weeks		n/a	WCR	
		iNSC -> iOL	FSK and VitC	N2, bFGF, PDGF-AA, T3	O4+ (30-36%)	~3 weeks			n/a	
		iNSC -> iA	n/a	FBS, N2, B27	GFAP+ (20-24%)					
Wei et al., 2020^(78)^	MEF	iNSC	CH, VPA, and Repsox	FBS, bFGF, EGF, Lif, IL-6, and FGF-5	Nestin+	12 days	ICC and FC	n/a	n/a	n/a
Tang et al., 2018^(79)^	MEF	iNSC	CH, VPA, and Repsox	Il-6, Fgf5, Lif, FBS, N2, B27, bFGF, and EGF	Nestin+ (~22% ), Sox2+, Pax6+, and Ascl1+	12 days	ICC	RT-qPCR, RNA-Seq, ATAC-seq, GREAT, siRNA knockdown	n/a	n/a
	TTF				Nestin+ (~8%), Sox2+, Pax6+, and Ascl1+			n/a		
Rujanapun et al., 2019^(66)^	HFF	iNSC	1-AZA, 5-AZA, DAPT and RA	FBS	TUJ1+ (>80%), NESTIN+, SOX2+, and PAX6+	5 days	ICC and MDC staining	RT-PCR, ROS	n/a	n/a
Cheng et al., 2014^(73)^	MEF and TTF	iNPC	(CH, VPA, Repsox,,, and VitC) or (NaB, LiCl and SB) or (TSA, Li2CO3 and Tranilast)	FBS, LIF, bFGF, EGF	Nestin (40%), Sox2 (50%), and Pax6 (60%)	~20 days	ICC and ALP	RT-qPCR, qPCR, Microarray, GO	n/a	ciNPCs are differentiated to neural lineage cells *in vivo* with no teratoma formation 1 month after transplantation.
		iNPC -> iN	VitC	N2, B27, BDNF, GDNF, IGF-1, cAMP	Tuj1 (~80%) and MAP2+	7 days		n/a	WCR	n/a
		iNPC -> iOL	n/a	N2, B27; bFGF, PDGF-AA, T3	Olig2+/Mbp+ (~25%)	~12 days			n/a	
		iNPC -> iA		N2, B27, BMP4, FBS	GFAP+ (~90%)	7 days				
	HUC	iNPC	CH, VPA, Repsox	n/a	Sox2+, Nestin+, Sox1+ and Pax6+	~20 days		RT-qPCR		
		iNPC -> iN	VitC	N2, B27, BDNF, GDNF, IGF-1, cAMP	Tuj1+/MAP2+	~14 days		n/a		
		iNPC -> iA			GFAP+	~30 days				
Chen et al., 2021^(82)^	SCAP	iNPC	CH, VPA, Repsox, FSK, SP600125, GO6983, and Y-27632	N2, B27, bFGF, and cAMP	Nestin+, Pax6+, and Sox2+	3 days	ICC	RT-qPCR	Cell proliferation assay	n/a
		iNPC -> iN			NFM+, NeuN+, and MAP2+	~4 days		RT-qPCR and western blot	WCR and cell proliferation assay	
Pan et al., 2021^(74)^	MEF	iNCC	CH, VPA, SB, RepSox, LDN, Y-27632, RA, FSK, A83-01, EPZ, RG108, 5-Aza, SMER28, AM580, and Parnate	N2, B27, bFGF, EGF, and BMP4	P75+, HNK1+, AP2ɑ+, and Nestin+	~12 days	ICC and TEM	RT-PCR and RNA-seq	Cell proliferation assay	MEF-derived ciNCCs are further differentiated into induced corneal endothelial cells and grafted in the rabbit corneal endothelial dysfunction model, showing the capacity to reverse the corneal opacity indicating their therapeutic effect for corneal endothelial deficiency.
		iNCC -> iN	VitC and db-cAMP	BDNF, GDNF, and NT3	Tuj1+ and Peripherin+	10-20 days	ICC	n/a	WCR	
		iNCC -> iSC	db-cAMP	N2, B27, CNTF, and neuregulin	GFAP+ and S100B+	2-4 weeks				
Hosseini Farahabadi et al., 2020^(80)^	HFF	iNCC	DM and TSA	FBS, N2, B27, BDNF, GDNF, CNTF	PAX6+ (~24%)	12 days	ICC and FC	qPCR	n/a	n/a
Sotthibundhu et al., 2022^(81)^	HFF	Neural cells	CH, VPA, FSK, SP600625 and Y-27632	Melatonin, BDNF, GDNF, and NT3	BRN2+, ASCL1+, MYT1L+, TUJ1+, DCX+, SOX2+, and NEUN+	14 days	ICC	RT-qPCR and western blot	n/a	n/a
Heng et al., 2019^(91)^	SCAP and DPC	Neural cells	CH, VPA, Repsox, FSK, SP600125, GO6983, Y-27632, and DM	N2, B27, cAMP, and bFGF	NeuN+, NFM+, NSE+, and MAP2+	14 days	ICC	RT-qPCR and western blot	Fluo-4 AM calcium flux assay	n/a
Samoilova et al., 2019^(58)^	DPC	Neural cells	CH, VPA, RG108, А83-01, DM, Tzv, VitC, FSK, and ISX9	B27, bFGF	MEF2C+, ASCL1+, POU3F2+, GFAP+, and SOX2+	21 days	ICC and FC	RT-PCR	n/a	n/a
Takayama et al., 2017^(47)^	MEF	iN	CH, VPA, Repsox, FSK, Parnate, DM, SB, RA, and VitC	FBS, bFGF, Lif, BDNF, GDNF	TUJ1+, synapsin-1+, MAP2+, and NeuN+	~19 days	ICC	RT-qPCR, Microarray	Ca2+ imaging	n/a
Li et al., 2015^(86)^	MEF	iN	CH, FSK, ISX9 and I-BET151	bFGF, BDNF and GDNF	TUJ1+ (~90%), TAUEGFP+/TUJ1+ (71%) and NEUN+/TUJ1+ (30%)	16-20 days	ICC	RT-qPCR	WCR	n/a
He et al., 2015^(85)^	MEF	iN	VitC	bFGF, N2, Lif, and βMe	TuJ1+ (46.3%)	16 days	ICC and FC	qPCR and RNA-seq	WCR	Neuronal-like cells are capable of survival after being transplanted into the mouse brain.
Hu et al., 2019^(65)^	MEF and HFF	iN	CH, VPA, FSK, LDN, SB, SP600125, and Y-27632	FBS, BDNF, GDNF, and NT3	Tuj1+ (87.03%)	14 days	ICC and FC	qPCR	cMEP	iNs lead to significant nerve regeneration and functional recovery on SCI rats transplantation.
Qin et al., 2018^(87)^	MEF and HFF	iN	CH or Kenpaullone, FSK, Y- 27632, PUR, and RA	FBS, B27, BDNF, GDNF, NT3	TUJ1+/HB9+ (~90%) and TUJ1+/ISL1+ (~90%)	3-5 days	ICC	RT-qPCR	n/a	Neuronal conversion *in vivo* could convert resident cells into cells expressing TUJ1 and motor neuron markers HB9, ISL1, and CHAT after 2 days of implantation of small molecule-soaked beads.
Wan et al., 2018^(88)^	HFF	iN	CH, VPA, DMH1, Repsox, FSK, Y-27632, and SP600125	cAMP, N2, B27, BDNF, GDNF, NT3	TUJ1+/MAP2+ (~65%)	7-14 days	ICC and FC	RT-qPCR	n/a	n/a
Hu et al., 2015^(43)^	HFF	iN	CH, VPA, RepSox, FSK, SP600125, GO6983, and Y-27632	BDNF, GDNF, NT3, and CFD	Tuj1+/Map2+ (~20%), Dcx+, NeuN+, and vGLUT1+	21-28 days	ICC and FC	RT-qPCR	WCR	n/a
Dai et al., 2015^(53)^	HFF	iN	CH, SB, LDN, PD, Pifithrin-α, and FSK	N2, B27	Tuj1+ (88.2% ± 3.9%) and MAP2+	21 days	ICC	n/a	n/a	n/a
Yang et al., 2020^(89)^	HFF	iN	CH, FSK, RepSox, SP600125, GO6983, Y-27632, IXS9, and I-BET151	N2, B27, cAMP, bFGF, BDNF, GDNF, NT3	Tuj1+, Map2+, and GAPDH+	30 days	ICC, mitochondrial staining, and TEM	RT-qPCR and western blot	WCR	n/a
Xu et al., 2019^(57)^	HUC	iN	CH, VPA, A83-01, NaB, Y-27632, TTNPB, and FSK	N2, B27, FGF, EGF, HGF	Tuj1+/MAP2+ (38.36% )	12 days	ICC	qPCR	WCR	n/a
Liu et al., 2020^(68)^	HUC	iN	CH, VPA, Repsox, FSK, SP600625, GO6983, Y-27632, I-BET151, ISX9, RA, QVD-OPH, and Vit C	N2, B27, cAMP-Na, BDNF, GDNF, IGF, and NT3	Tuj1+, MAP2+, Tau+, PSA-Ncam+, GABA+, NeuN+, and SYNJ1+	14 days	ICC	RT-qPCR	WCR	n/a
Zou et al., 2022^(90)^	SCAP	iN	CH, FSK, and DM	BDNF and gelatin methacrylate hydrogel	Tuj1+ and MAP2+	3-7 days	ICC	RT-qPCR and western blot	Cell proliferation assay	n/a
Yang et al., 2019^(55)^	HFF	iGlN	CH, LDN, RG108, DM, P7C3-A20, A83-01, ISX9, FSK, Y-27632, DAPT, PD, and PUR	FBS, N2, B27, BDNF, GDNF, IGF-1, and NT-3	Tuj1+ (76%)	14 days	ICC	qPCR, RT-PCR, RNA-Seq	WCR	iNs transplanted into the mouse brain could successfully survive *in vivo* and integrate into resident circuits.
Qin et al., 2020^(92)^	HFF	DA iN	VPA, Repsox, kenpaullone, FSK, PUR, SHH, and VitC	FGF-8b, bFGF, N2, B27, Wnt1, Wnt5, BDNF, and GDNF	TUJ1+/TH+ (87.88%)	13-22 days	ICC	RT-qPCR	WCR	n/a
Wilson et al., 2018^(67)^	hEPI-NCSC	iPNSN	CH, SHH, LDN, and DAPT	NT3, FBS	TRPV1+, SP+, and CGRP+	18 days	ICC	qPCR	Ca2+ imaging	n/a
Han et al., 2017^(93)^	MEF	iA	CH, Repsox, Parnate, FSK, VPA, and A83-01	FBS, bFGF	GFAP+	~22 days	ICC	sc-qPCR, qPCR, Microarray, PPIN, WGCNA, GO, ChIP-seq, and DNA methylation	WCR	n/a
E. Tian et al., 2016^(21)^	MEF, TTF	iA	CH, VPA, SB, Parnate, and OAC1	FBS, FGF	Gfap+ (~38%), S100b+, and Aldh1l1+	20-25 days	ICC	qPCR, RT-PCR, Microarray, GO	Ca2+ imaging; Glutamate Uptake Assay	iAs can survive engraftment and maintain astrocytic marker expression *in vivo*.
	HFF			FBS, FGF, N2, B27, CNTF	Gfap+ (>15%) and S100b+ (>40%)	~50 days				
Thoma et al., 2014^(94)^	HFF	iSC	VitC, SHH, noggin, SB, CP21, Compound B, and db-cAMP	FBS, bFGF, EGF, N2, B27, BDNF, GDNF, Dll4, Jagged1, and VitA	PLP+, GalC+, Krox-20+, and S100B+	~39 days	ICC and FC	n/a	n/a	n/a
Liu et al., 2019^(84)^	MEF	iOPC	M9 (CH, LDN, A83-01, RA, Hh-Ag1.5, RG108, Parnate, SMER28, and bFGF), SHH and Tzv	EGF, FBS, BSA, N2, B27, and PDGF-AA	Olig2+ (∼24.72%) and Nkx2.2+ (12.88%)	14 days	ICC and FC	RT-qPCR and RNA-seq	Myelination assay	n/a
		iOPC -> iOL	SHH, LDN, and db-cAMP	T3 and NT3	O4+, Olig2+, MBP+, MAG+, and MOG+	8-12 days	ICC	RT-qPCR		
		iOPC -> iA	Basal medium	FBS	GFAP+ (~1.12%)	8-12 days			n/a	

Donor cells: MEF: mouse embryonic fibroblast; HUC: human urinary cell; SCAP: stem cells from the apical papilla; TTF: tail-tip fibroblast; HFF: Human fibroblast; DPC: dental pulp cell; hEPI-NCSC: human epidermal neural crest stem cells. Cell lineages achieved: iGN: induced GABAergic neuron; iGlN: induced glutamatergic neurons; iDPC: induced dopaminergic neural progenitor cell; iOPC: induced oligodendrocyte progenitor cell; iN: induced neuron; DA iN: induced dopaminergic neuron; iOL: induced oligodendrocyte; iA: induced astrocyte; iNCC: induced neural crest lineage cell; iNSC: induced neural stem cell; iAPC: induced astroglial progenitor cell; iNPC: induced neural progenitor cell; iSC: induced Schwann cell; iPNSN: induced peptidergic nociceptive sensory neurons. Small molecules: CH: CHIR99021; SB: SB431542; PD: PD0325901; LDN: LDN193189; FSK: forskolin; PUR: purmorphamine; EPZ: EPZ004777; DAPT: γ-secretase inhibitor; 1-AZA:, 1-azakenpaullone; SHH: human Sonic hedgehog; RA: retinoic acid; ISX9: isoxazole9; VitC: ascorbic acid; NaB: sodium butyrate; DM: dorsomorphin; SP: sodium pyruvate; TSA: trichostatin; Tzv: thiazovivin; LiCl: lithium chloride; L_i_2CO_3_: lithium carbonate. Supplementation: Fgf5: fibroblast growth factor 5; dbcAMP: dibutyryl-cAMP; PDGF-AA: platelet-derived growth factor-AA; FGF-8b: fibroblast growth factor-8b; bFGF: basic fibroblast growth factor; HGF: hepatocyte growth factor; Il-6: interleukin-6; BDNF: brain-derived neurotrophic factor; GDNF: glial cell-derived neurotrophic factor; CNTF: ciliary neurotrophic factor; NM: neurobasal medium; Lif: leukemia inhibitory factor; FGF2: human fibroblast growth factor 2; hBDNF: human brain derived neurotrophic factor; T3: triiodothyronine; PDGF-AA: platelet-derived growth factor AA; FBS: fetal bovine serum; FCS: fetal calf serum; NT3: neurotrophin 3; CNTF: ciliary neurotrophic factor; CFD: complement factor D; BSA: bovine serum albumin; βMe: β-mercaptoethanol; IGF-1: insulin-like growth factor 1; Dll4: delta-like 4. Phenotype analysis: ICC: immunocytochemistry; FC: flow cytometry; MDC staining: monodansylcadaverine staining; TEM: transmission electron microscopy; ALP: alkaline phosphatase analysis. Transcript analysis: sc-qPCR: single-cell quantitative polymer chain reaction; ChIP-seq: chromatin immunoprecipitation sequencing; ATAC-seq: transposase-accessible chromatin using sequencing; PPIN: protein-protein interaction network analysis; GO: Gene ontology analysis; ROS: reactive oxygen species investigation; WGCNA: weighted correlation network analysis; GREAT: Genomic Regions Enrichment of Annotations Tool; siRNA knockdown: small interfering RNA knockdown. Functional analysis: WCR: whole-cell patch-clamp recordings; cMEP: cortical motor-evoked potential.

The combination of the SMs VPA, A83-01, Tzv, and PUR was also capable to convert MEFs into Nestin+/Sox2+ iNSCs, in 12 days, similar to NSCs in terms of morphology and self-renewal property.^(77)^Furthermore, these iNSCs differentiated into oligodendrocytes, astrocytes, and different types of mature functional neurons (GABAergic, dopaminergic, and cholinergic) *in vitro*. In the same way, the molecules LDN, SB, CHIR, VPA, DAPT, SHH, and PUR, applied at different time points, were able to directly reprogram MEFs into Nestin+/Sox2+ iNSCs, in 10 days.^(63)^ The iNSCs were able to differentiate into GFAP+ astrocytes, Olig2+ oligodendrocytes, and Tuj1+ neurons when treated with EGF and FGF-free NSC culture media. Similarly, Wei et al. described a protocol for MEF-derived iNSCs induced by the chemical cocktail CHIR, VPA, and RepSox, and the ciNSCs expressed Nestin + within 12 days of induction.^(78)^

Pan et al. used CH, VPA, SB, RepSox, LDN, Y-27632, RA, FSK, A83-01, EPZ, RG108, 5-Aza, SMER28, AM580, and parnate to generate P75+, HNK1+, AP2ɑ+, and Nestin+ induced neural crest cells (iNCCs) from MEFs in approximately 12 days. The iNCCs were further differentiated into Tuj1+/Peripherin+ iNs and GFAP+/S100B+ iSCs. Notably, iNCCs were also capable of differentiating into induced corneal endothelial cells (ciCECs) and were grafted into a rabbit corneal endothelial dysfunction model, showing the capacity to reverse corneal opacity, thereby indicating their therapeutic effect.^(74)^

Cocktails that induce cell transdifferentiation can be composed of SMs alone or in combination with other molecules such as growth factors (GFs), proteins, or self-replicating mRNAs.^(64)^ Growth factors promote both cell conversion and maturation by modulating signaling pathways.^(52)^ Tang et al.^(79)^ showed the generation of MEF-derived and tail-tip fibroblast (TTF)-derived iNSCs that were Nestin+, Sox2+, Pax6+, and Ascl1+ using VPA, CHIR, and RepSox in combination with the GFs interleukin-6 (Il-6), leukemia inhibitory factor (Lif), and fibroblast growth factor 5 (Fgf5) for 12 days, without the introduction of exogenous genes or procedures that lead to cellular physical stress.

Cheng et al. converted MEFs, TTFs, and epithelial cells derived from the human urinary cells (HUCs) into iNPCs.^(73)^ Three chemical cocktails were tested under physiological hypoxic culture conditions (5% O_2_) during the first 20 days: VCR (VPA, CHIR, and RepSox), NLS (NaB, LiCl, and SB), and TLT (trichostatin [TSA], Li2CO3, and tranilast). The ciNPCs showed morphological and gene expression characteristics of NPCs, as well as the ability to further differentiate into neural lineages. In addition, ciNPCs differentiated into neural lineage cells *in vivo* with no teratoma formation one month after transplantation into the mouse brain.

Human cells were successfully transdifferentiated into iNSCs using the SMs. Hosseini Farahabadi et al.^(80)^ promoted the induction of HFFs into induced neural crest PAX6+ cells using DM and TSA for 12 days. HFFs were also induced into TUJ1+, NESTIN+, SOX2+, and PAX6+ iNSCs using a cocktail of 1-AZA, 5-AZA, RA, and DAPT for five days.^(66)^ Moreover, Sotthibundhu et al.^(81)^ obtained HFF-derived neural cells that showed BRN2+, ASCL1+, MYT1L+, TUJ1+, DCX+, SOX2+, and NEUN+ after 14 days of induction with CH, VPA, FSK, SP600625, and Y-27632 and supplemented with melatonin, BDNF, GDNF, and NT3.

Human dental pulp cells (DPCs), an easily collectable cell type, were also induced to neuroglial lineage cells using a cocktail composed by VPA, RG108, A83-01, DM, Tzv, CHIR, FSK, and Isx9 for 21 days.^(58)^ The neural cells showed immunophenotypic and genetic signals of neural stem cells but were not capable of adequate terminal differentiation. It was suggested that the addition of gene expression modifier factors might be required to allow the reproducible generation of human neural progenitor cells capable of generating neural tissue for regenerative therapy.

Chen et al.^(82)^ applied the cocktail CH, VPA, RepSox, FSK, SP600125, GO6983, and Y-27632 to achieve Nestin+, Pax6+, and Sox2+ iNPCs transdifferentiated from stem cells from the apical papilla (SCAPs) in three days and NFM+, NeuN+, and MAP2+ functional iN after four days of treatment.

Finally, several studies showed that somatic cells can also be reprogrammed into oligodendrocyte precursor cells (OPCs) with a capability of being further differentiated into myelin-generating cells both *in vitro* and *in vivo*.^(83)^ A combination of CHIR, RA, Hh-Ag1.5, RG108, LDN, A83-01, SMER28, parnate, SHH, Tzv, and bFGF was shown to be capable of directly converting MEFs into Olig2+/Nkx2.2+ chemically induced oligodendrocyte precursor cells (ciOPC).^(84)^ It was also shown that these cells have morphology, gene expression, and self-renewal capacity similar to those of OPC-derived neural stem cells. In addition, these ciOPCs differentiated into functional oligodendrocytes that generate myelin around the axons *in vitro*.

## Chemically induced neuronal and glial lineage cells Chemically induced neurons

Somatic cell conversion by SMs can generate not only neural stem or progenitor cells but also terminally differentiated cells (Table 2). A direct cell conversion protocol using bFGF, N2 supplement, Lif, VitC, and β-mercaptoethanol (βMe) for 16 days induced MEFs into TuJ+ iNs that were capable of surviving after transplantation into mouse brains.^(85)^ The use of a cocktail composed of FSK, ISX9, CHIR, SB, and I-BET151 also enabled the conversion of MEFs into TUJ1+ ciNs after approximately 16 days of induction.^(86)^ After ciNs maturation, action potentials and functional synapse formations were observed.

Mouse embryonic fibroblasts-derived iNs were also obtained using CH, VPA, RepSox, FSK, parnate, DM, SB, RA, and VitC for approximately 19 days.^(47)^ The iNs were TUJ1+, synapsin-1+, MAP2+, and NeuN+ and displayed calcium influx properties. This study also showed that iNs pass through a neural crest precursor stage, a stage in which cells can differentiate into neural crest lineage cells, such as osteocytes, adipocytes, smooth muscle cells, and sympathetic neurons.

Other studies have used SMs to directly induce neuronal conversion in human cells. For instance, CH and kenpaullone, when combined with FSK, Y- 27632, PUR, and RA, can be efficiently used to directly convert MEFs and TFFs into TUJ1+/HB9+ ciNs, both *in vitro* and *in vivo*.^(87)^ Additionally, *in vivo* implantation of SM-soaked beads converted the resident cells into TUJ1+ ciNs, HB9+, ISL1+, and CHAT+ motor neurons after two days.

Another study used a combination of CH, VPA, FSK, LDN, SB, SP600125, and Y-27632 to induce the transformation of HFFs and MEFs into Tuj1+ ciNs within 14 days.^(65)^ Murine or human ciNs embedded in three dimensional (3D) silk fibrous materials and transplanted into rat sectioned spinal cord stumps showed the capacity to promote considerable nerve regeneration and functional recovery in rats with spinal cord injury after eight weeks.

The application of VPA, CHIR, RepSox, FSK, SP600125, GO6983, and Y-27632 to HFFs resulted in their direct conversion into Tuj1+/Map2+, Dcx+, NeuN+, and vGLUT1+ ciNs.^(43)^ These ciNs resembled hiPSC-derived neurons and human TF-induced iNs in many respects, such as morphology, gene expression profiles, and functional properties. This protocol was further applied to induce ciNs in patients with familial Alzheimer’s disease, therefore providing an alternative strategy for regenerative therapies and studying neurological diseases. Another study demonstrated that human lung fibroblasts could be converted directly into ciNs using VPA, CHIR, DMH1, RepSox, FSK, Y-27632, and SP600125 over a period of 7-14 induction days.^(88)^ After an additional maturation period, these ciNs expressed the neuron-specific gene Tuj1+/Map2+ and exhibited neuronal morphology. HFFs-derived iNs were also generated after 21 days of treatment with CH, SB, LDN, PD, pifithrin-α, and FSK resulting in Tuj1+/MAP2+ iNs.^(53)^ Furthermore, the cocktail CH, RepSox, FSK, GO6983, SP600125, Y-27632, IXS9, and I-BET151 converted HFFs into functional Tuj1+, Map2+, and GAPDH+ iNs in 30 days.^(89)^

In addition, HUCs can be partially converted into neuron-like cells after 14 days of chemical induction, showing the expression of neuron-specific genes, such as Tuj1, MAP2, Tau, PSA-Ncam, NeuN, and SYNJ1.^(68)^ The application of CHIR, VPA, A83-01, NaB, Y-27632, TTNPB, and FSK also generated HUC-derived Tuj1+/MAP2+ ciNs with typical neuronal morphology, gene expression, and electrophysiological properties on Day 12 after induction.^(57)^

Tuj1+/MAP2+ iNs were also obtained from SCAPs by adding CH, FSK, and DM to BDNF and gelatin methacrylate hydrogels for 3-7 days.^(90)^ It was also demonstrated that NeuN+, NFM+, NSE+, and MAP2+ cells could be obtained from SCAPs using CH, VPA, RepSox, FSK, SP600125, GO6983, Y-27632, and DM for 14 days.^(91)^ In this study, DPC-derived neural-like cells were also obtained, and the Fluo-4 AM Calcium Flux Assay demonstrated that these cells exhibited consistently higher calcium transient peaks (F/Fo) compared to that of the controls.

Specifically, chemically induced neuron types were also obtained from somatic cells. HFFs were converted into induced glutamatergic neurons (iGlNs) that expressed as Tuj1+ in 10 days by combining CH, LDN, RG108, DM, P7C3-A20, A83-01, ISX9, FSK, Y-27632, DAPT, PD, and PUR.^(55)^ The iGlNs survived for at least two months and showed functional activity when co-cultured with astrocytes. Furthermore, after transplantation into the mouse brain, iGlNs survived and integrated into resident circuits *in vivo*.^(55)^ HFF-derived dopaminergic neurons (DA-iNs), TUJ1+ / TH +, capable of firing single action potentials, were obtained using the cocktail of VPA, RepSox, kenpaullone, FSK, PUR, SHH, and VitC plus the factors FGF-8b, bFGF, N2, B27, Wnt1, Wnt5, BDNF, and GDNF.^(92)^ Finally, using CH, SHH, LDN, and DAPT for 18 days, human TRPV1+, SP+, and CGRP+ peptidergic nociceptive sensory neurons were generated from human epidermal neural crest stem cells (hEPI-NCSCs), which are multipotent somatic stem cells located in the bulge of hair follicles.^(67)^

## Chemically induced astrocytes

GFAP+ chemically induced astrocytes (ciAs) were obtained from MEFs approximately 22 days after the application of CH, RepSox, parnate, FSK, VPA, and A83-01.^(93)^ In this protocol, MEFs first went through a multilineage state (iMT), and according to the different chemical combinations applied, it was possible to reach myocytic, glial, or adipocytic lineages.

The addition of a cocktail composed of VPA, CHIR, SB, parnate, and OAC1 reprogrammed MEFS into functional GFAP+, S100b+, and Aldh1l1+ astrocytes after 20-25 days.^(38)^ The ciAs can promote neuronal maturation, synaptic formation, glutamate uptake, and induction of calcium influx in response to glutamate stimulation. In addition, after engraftment in the lateral ventricles of immunodeficient neonatal non-obese diabetic mice, these cells maintained astrocytic marker expression *in vivo*. The same cocktail was also tested on HFFs, which produced astroglial progenitor cells that further differentiated into functional GFAP+ and S100b+ astrocytes.

## Chemically induced schwann cells

SMs have also been tested for the direct conversion of HFFs into Schwann cells.^(94)^ The two-step protocol containing Vit C, SHH, noggin, SB, CP21, Compound B, and dibutyryl-cAMP (db-cAMP) led the cells to reach a transient neural precursor stage that later differentiated into induced Schwann cells (iSCs) after approximately 39 days of induction. The iSCs expressed specific markers, such as PLP, GalC, Krox-20, and S100B, and demonstrated neuroprotective and myelination capacities *in vitro*.

## DISCUSSION

Cell transdifferentiation is a faster and safer way to obtain the desired cells than iPSC reprogramming followed by cell differentiation. The use of SMs as potential tools to promote cell transdifferentiation is of particular interest owing to their stability and affordability. To date, several attempts have been made to induce neural transdifferentiation from murine and human somatic cells using SMs, showing a promising way to generate neural progenitor cells, neurons, and glial cells with potential research and clinical applications.

However, the use of SMs as direct conversion inducers is still in its infancy and some limitations need to be addressed. Different protocols have shown variable cell conversion efficiencies, which may be related to the SM cocktail used. In addition, it is important to keep in mind that many chemically induced neuronal transdifferentiation protocols have been developed using mouse cells and that they may not necessarily work for human cells due to species differences, thus needing further adjustments for better conversion efficiency for humans.^(95)^ Other factors can also influence the effectiveness of cell conversion, as well as the viability and functionality of the cells produced by these chemically induced neural transdifferentiation protocols. One such factor might be the age of the donor; the use of older cell populations can reduce transdifferentiation efficiency owing to the accumulation of somatic mutations or epigenetic status.^(94)^ In the same way, the cell source might facilitate transdifferentiation efficiency, as some cell types may present an epigenetic landscape more closely related to the targeted cell or more prone to manipulation in the desired direction. In this regard, multipotent stem cells found in different tissues, such as hair follicles and dental pulp, deserve special attention because they are known to express neuronal markers.^(58,67,96)^

In addition, some environmental conditions to consider as possible influencers of cell conversion efficiency towards a neuronal fate are cell-cell contacts, paracrine or autocrine signaling, and factors secreted by the cells. These aspects can be manipulated in cell cultures using different extracellular matrices, two dimensional (2D) or 3D cultures, or different media regimens.^(95)^ In contrast, *in vivo* systems might be an interesting alternative approach because reprogramming under these conditions has been shown to be more efficient and capable of inducing more mature cells than *in vitro* reprogramming.^(95)^ The use of SMs in combination with other approaches may reveal many possibilities for establishing more efficient transdifferentiation protocols.

The use of microRNAs (miRNAs), ^(92-94)^ low-intensity ultrasound (LIUS),^(97)^ and special biophysical surfaces, such as microgrooved surfaces^(69)^ has been associated with better efficiencies in cell conversion. In addition, studies have shown that the microenvironment offered by 3D cultures can optimize cell conversion induced by defined factors.^(56,98)^ Thus, it would be interesting to test these strategies in combination with SMs to promote neural transdifferentiation.^(99)^

Another important factor to consider in an attempt to increase the efficiency of the neural conversion process is the use of hypoxia in cell culture, as neural cells naturally reside in hypoxic niches of the central nervous system, where cell proliferation and differentiation occur. Hypoxia may have a beneficial effect on neural transdifferentiation^(73)^ although the underlying mechanisms behind that still require further elucidation. It would be interesting to understand these mechanisms to test possible compounds that can replace hypoxia.^(100)^

Interestingly, molecules with antioxidant properties, such as vitamin E, nicotinate, vitC, resveratrol, N-acetylcysteine, EUK134, ebselen, mito-TEMPO, and NADPH oxidase inhibitors, can help in cell reprogramming and differentiation. It has been observed that these antioxidants help in the conversion of fibroblasts to iPSCs, the differentiation of iPSCs into target cells, and the direct conversion of fibroblasts into target cells^(101)^ placing these molecules as promising candidates for neural transdifferentiation. Another class of molecules that deserves to be tested are nuclear receptor agonists and antagonists of SMs. These molecules are known to aid in cell reprogramming or induce neural differentiation but have not been tested in transdifferentiation protocols.

An efficient approach for identifying potential drugs to further improve neural transdifferentiation protocols is to search for pathways that are differentially regulated during this process. For instance, a study that used meta-analysis and regulatory gene network analysis tools to explore gene expression data identified gene regulatory components related to the direct conversion of fibroblasts into nerve cells. The results of that study indicate that miR-9, miR-30, and the TFs JUN, SP1, TP53, MYC, and SMAD2 are central regulatory elements in the process of cell conversion.^(102)^ This type of data can help identify molecules that interact with key components associated with greater conversion efficiency and specificity. Furthermore, the identification and suppression of master genes associated with the native or somatic states of different cell types can contribute to increasing the efficiency and fidelity of direct conversion.^(103)^

However, the mechanisms underlying the action of SMs on neural transdifferentiation require further elucidation.^(104)^ Some SM-mediated actions are nonspecific and a specific SM can have multiple targets, making it challenging to interpret its effects. Furthermore, toxicity and unexpected side effects in humans represent challenges for the clinical application of transdifferentiation protocols based on the use of SMs, especially *in vivo*. Another challenge is to establish efficient methods for delivering chemical compounds into the desired cell niches.^(105)^ In contrast, sophisticated pharmacological approaches might be used to identify optimal concentrations, exposure times, dose responses, and synergistic effects in systematic, high-throughput assays, helping to circumvent these issues.^(71,106)^

## CONCLUSION

Although much remains to be elucidated, there is substantial evidence showing the potential of small molecules, either alone or in conjunction with other approaches, for neuronal transdifferentiation. Thus, further efforts are needed to improve conversion efficiencies and test the safety of small molecule-based protocols that can be used for the generation of neuronal disease modeling platforms and for *ex-vivo* or *in vivo* regenerative therapy applications.
