# Supplementary material for: Chemical transdifferentiation of somatic cells to neural cells: a systematic review
Source: Einstein (Sao Paulo). 2024 Nov 12;22:eRW0423. doi: 10.31744/einstein_journal/2024RW0423 (PMC11634374; doi:10.31744/einstein_journal/2024RW0423)
Supplement: Supplementary file 1 [file 2317-6385-eins-22-eRW0423-suppl01.pdf]

SUPPLEMENTARY MATERIAL

Chemical transdifferentiation of somatic cells to neural cells: a systematic review

Paulo Victor Visintin, Bruna Lancia Zampieri, Karina Griesi-Oliveira

DOI: 10.31744/einstein_journal/2024RW0423

Table 1S. Search strategies used in databases

Pubmed - Search strategy carried out on December 1, 2021 - 227 results	("cell transdifferentiation"[MeSH Terms] OR "cell dedifferentiation"[MeSH Terms] OR "cellular reprogramming"[MeSH Terms] OR "cellular reprogramming techniques"[MeSH Terms] OR "cell dedifferentiation"[Title/Abstract] OR "cellular dedifferentiation"[Title/Abstract] OR "cell reprogramming"[Title/Abstract] OR "cellular reprogramming"[Title/Abstract] OR "transdifferentiation"[Title/Abstract] OR "cell generation"[Text Word] OR "direct conversion"[Text Word] OR "direct reprogramming"[Text Word] OR "direct cell reprogramming"[Text Word] OR "direct lineage reprogramming"[Text Word] OR "direct differentiation"[Text Word] OR "lineage conversion"[Text Word] OR "cell induction"[Text Word] OR "neural induction"[Text Word] OR "neural conversion"[Text Word]) AND ("small molecule libraries"[MeSH Terms] OR "small molecule"[Title/Abstract] OR "small molecules"[Title/Abstract] OR "chemical cocktail"[Title/Abstract] OR "chemical cocktails"[Title/Abstract] OR "chemical induction"[Title/Abstract] OR "chemical approach"[Title/Abstract] OR "chemical approaches"[Title/Abstract] OR "integration free"[Text Word] OR "non integrative"[Text Word]) AND ("neural stem cells"[MeSH Terms] OR "neurons"[MeSH Terms] OR "neurogenesis"[MeSH Terms] OR "neuroglia"[MeSH Terms] OR "neural stem cell"[Title/Abstract] OR "neural stem cells"[Title/Abstract] OR "neural progenitor cell"[Title/Abstract] OR "neuron"[Title/Abstract] OR "neurons"[Title/Abstract] OR "neural"[Title/Abstract] OR "neurogenesis"[Text Word] OR "ectoderm*"[Text Word] OR "neuroectoderm*"[Text Word] OR "nsc"[Text Word] OR "nscs"[Text Word] OR "insc"[Text Word] OR "npc"[Text Word] OR "npcs"[Text Word] OR "inpc"[Text Word] OR "nplc"[Text Word] OR "microglia"[Title/Abstract] OR "astrocytes"[Title/Abstract] OR "oligodendrocytes"[Title/Abstract] OR "schwann cell"[Title/Abstract] OR "neuroglia*"[Text Word] OR "astroglia*"[Text Word] OR "glia*"[Text Word] OR "microglial"[Text Word] OR "OL cell"[Text Word])<br>https://pubmed.ncbi.nlm.nih.gov/?term=longqueryb81e9100e2bc4b0f7ca4&size=200
Scopus - Search strategy carried out on December 1, 2021 - 255 results	((TITLE-ABS-KEY ("cell dedifferentiation") OR TITLE-ABS-KEY ("cellular dedifferentiation") OR TITLE-ABS-KEY ("cell reprogramming") OR TITLE-ABS-KEY ("cellular reprogramming") OR TITLE-ABS-KEY (transdifferentiation) OR TITLE-ABS-KEY ("cell generation") OR TITLE-ABS-KEY ("direct conversion") OR TITLE-ABS-KEY ("direct reprogramming") OR TITLE-ABS-KEY ("direct cell reprogramming") OR TITLE-ABS-KEY ("direct lineage reprogramming") OR TITLE-ABS-KEY ("direct differentiation") OR TITLE-ABS-KEY ("lineage conversion") OR TITLE-ABS-KEY ("cell induction") OR TITLE-ABS-KEY ("neural induction") OR TITLE-ABS-KEY ("neural conversion"))) AND ((TITLE-ABS-KEY ("small molecule") OR TITLE-ABS-KEY ("small molecules") OR TITLE-ABS-KEY ("chemical cocktail") OR TITLE-ABS-KEY ("chemical cocktails") OR TITLE-ABS-KEY ("chemical induction") OR TITLE-ABS-KEY ("chemical approach") OR TITLE-ABS-KEY ("chemical approaches") OR TITLE-ABS-KEY ("integration free") OR TITLE-ABS-KEY ("non integrative"))) AND ((TITLE-ABS-KEY ("neural stem cell") OR TITLE-ABS-KEY ("neural stem cells") OR TITLE-ABS-KEY ("neural progenitor cell") OR TITLE-ABS-KEY (neuron) OR TITLE-ABS-KEY (neurons) OR TITLE-ABS-KEY (neural) OR TITLE-ABS-KEY (ectoderm) OR TITLE-ABS-KEY (ectodermal) OR TITLE-ABS-KEY (neuroectoderm) OR TITLE-ABS-KEY (neuroectodermal) OR TITLE-ABS-KEY (nsc) OR TITLE-ABS-KEY (nscs) OR TITLE-ABS-KEY (insc) OR TITLE-ABS-KEY (npc) OR TITLE-ABS-KEY (npcs) OR TITLE-ABS-KEY (inpc) OR TITLE-ABS-KEY (nplc) OR TITLE-ABS-KEY (astrocytes) OR TITLE-ABS-KEY (oligodendrocytes) OR TITLE-ABS-KEY ("schwann cell") OR TITLE-ABS-KEY (neuroglia) OR TITLE-ABS-KEY (neuroglial) OR TITLE-ABS-KEY (astroglia) OR TITLE-ABS-KEY (astroglial) OR TITLE-ABS-KEY (glia) OR TITLE-ABS-KEY (glial) OR TITLE-ABS-KEY (microglia) OR TITLE-ABS-KEY (microglial) OR TITLE-ABS-KEY ("OL cell")))<br>https://www.scopus.com/results/results.uri?sort=plf-f&src=s&sid=706e459e10f15d63693e0ceff2c50824&sot=a&sdt=a&sessionSearchId=706e459e10f15d63693e0ceff2c50824&origin=searchadvanced&editSaveSearch=&txGid=bb15033619cc801b30129f2ee61a0218
Embase - Search strategy carried out on December 7, 2021 - 267 results	('cell transdifferentiation'/exp/mj OR 'cell dedifferentiation'/exp/mj OR 'cell reprogramming technique'/exp/mj OR 'cell dedifferentiation':ti,ab,kw OR 'cellular dedifferentiation':ti,ab,kw OR 'cell reprogramming':ti,ab,kw OR 'cellular reprogramming':ti,ab,kw OR transdifferentiation:ti,ab,kw OR 'cell generation':ti,ab,kw OR 'direct conversion':ti,ab,kw OR 'direct reprogramming':ti,ab,kw OR 'direct cell reprogramming':ti,ab,kw OR 'direct lineage reprogramming':ti,ab,kw OR 'direct differentiation':ti,ab,kw OR 'lineage conversion':ti,ab,kw OR 'cell induction':ti,ab,kw OR 'neural induction':ti,ab,kw OR 'neural conversion':ti,ab,kw OR 'neural induction'/exp/mj OR 'cell induction'/exp/mj) AND ('small molecule'/exp/mj OR 'chemical compound'/exp/mj OR 'small molecule':ti,ab,kw OR 'small molecules':ti,ab,kw OR 'chemical cocktail':ti,ab,kw OR 'chemical cocktails':ti,ab,kw OR 'chemical induction':ti,ab,kw OR 'chemical approach':ti,ab,kw OR 'chemical approaches':ti,ab,kw OR 'integration free':ti,ab,kw OR 'non integrative':ti,ab,kw) AND ('neural stem cell'/exp/mj OR 'nerve cell'/exp/mj OR 'glia cell'/exp/mj OR 'glial precursor cell'/exp/mj OR 'neural stem cell':ti,ab,kw OR 'neural stem cells':ti,ab,kw OR 'neural progenitor cell':ti,ab,kw OR neuron:ti,ab,kw OR neurons:ti,ab,kw OR neural:ti,ab,kw OR neurogenesis:ti,ab,kw OR ectoderm:ti,ab,kw OR ectodermal:ti,ab,kw OR neuroectoderm:ti,ab,kw OR neuroectodermal:ti,ab,kw OR nsc:ti,ab,kw OR nscs:ti,ab,kw OR insc:ti,ab,kw OR npc:ti,ab,kw OR npcs:ti,ab,kw OR inpc:ti,ab,kw OR nplc:ti,ab,kw OR microglia:ti,ab,kw OR astrocytes:ti,ab,kw OR oligodendrocytes:ti,ab,kw OR 'schwann cell':ti,ab,kw OR neuroglia:ti,ab,kw OR neuroglial:ti,ab,kw OR astroglia:ti,ab,kw OR astroglial:ti,ab,kw OR glia:ti,ab,kw OR glial:ti,ab,kw OR microglial:ti,ab,kw OR 'ol cell':ti,ab,kw)<br>https://www.embase.com/#advancedSearch/resultspage/history.1/page.1/25.items/orderby.date/source
Web of Science - Search strategy carried out on December 7, 2021 - 260 results	TS=("neural stem cell") OR TS=("neural stem cells") OR TS=("neural progenitor cell") OR TS=(neuron) OR TS=(neural) OR TS=(neurogenesis) OR TS=(ectoderm) OR TS=(ectodermal) OR TS=(neuroectoderm) OR TS=(neuroectodermal) OR TS=(NSC) OR TS=(iNSC) OR TS=(NPC) OR TS=(iNPC) OR TS=(NPLC) OR TS=(astrocytes) OR TS=(oligodendrocytes) OR TS=("schwann cell") OR TS=(neuroglia) OR TS=(astroglia) OR TS=(glia) OR TS=(microglia) OR TS=("OL cell")<br>TS=("small molecule") OR TS=("small molecules") OR TS=("chemical cocktail") OR TS=("chemical cocktails") OR TS=("chemical induction") OR TS=("chemical approach") OR TS=("chemical approaches") OR TS=("integration free") OR TS=("non integrative")<br>TS=(cell dedifferentiation) OR TS=("cellular dedifferentiation") OR TS=("cell reprogramming") OR TS=("cellular reprogramming") OR TS=(transdifferentiation) OR TS=("cell generation") OR TS=("direct conversion") OR TS=("direct reprogramming") OR TS=("direct cell reprogramming") OR TS=("direct lineage reprogramming") OR TS=("direct differentiation") OR TS=("lineage conversion") OR TS=("cell induction") OR TS=("neural induction") OR TS=("neural conversion")<br>https://www.webofscience.com/wos/woscc/summary/a3120a30-3dd4-48a9-abe8-f58403d525f5-21052ca9/relevance/1
